# Geographical Inequalities in Surgical Treatment for Localized Female Breast Cancer, Queensland, Australia 1997–2011: Improvements over Time but Inequalities Remain

**DOI:** 10.3390/ijerph13070729

**Published:** 2016-07-19

**Authors:** Peter D. Baade, Paramita Dasgupta, Philippa H. Youl, Christopher Pyke, Joanne F. Aitken

**Affiliations:** 1 Cancer Council Queensland, P.O. Box 201, Spring Hill, QLD 4004, Australia; paramitadasgupta@cancerqld.org.au (P.D.); pipyoul@cancerqld.org.au (P.H.Y.); joanneaitken@cancerqld.org.au (J.F.A.); 2 School of Mathematical Sciences, Queensland University of Technology, Gardens Point, Brisbane, QLD 4000, Australia; 3 Menzies Health Institute Queensland, Griffith University, Gold Coast Campus, Parklands Drive, Southport, QLD 4222, Australia; 4 School of Public Health and Social Work, Queensland University of Technology, Herston Road, Kelvin Grove, QLD 4059, Australia; 5 Mater Medical Centre, 293 Vulture Street, South Brisbane, QLD 4101, Australia; c_pyke@mc.mater.org.au; 6 School of Population Health, University of Queensland, Brisbane 4006, Australia; 7 Institute for Resilient Regions, University of Southern Queensland, Toowoomba 4350, Australia

**Keywords:** breast cancer, geography, inequalities, mastectomy, breast conserving surgery, socioeconomic

## Abstract

The uptake of breast conserving surgery (BCS) for early stage breast cancer varies by where women live. We investigate whether these geographical patterns have changed over time using population-based data linkage between cancer registry records and hospital inpatient episodes. The study cohort consisted of 11,631 women aged 20 years and over diagnosed with a single primary invasive localised breast cancer between 1997 and 2011 in Queensland, Australia who underwent either BCS (*n* = 9223, 79%) or mastectomy (*n* = 2408, 21%). After adjustment for socio-demographic and clinical factors, compared to women living in very high accessibility areas, women in high (Odds Ratio (OR) 0.58 (95% confidence intervals (CI) 0.49, 0.69)), low (OR 0.47 (0.41, 0.54)) and very low (OR 0.44 (0.34, 0.56)) accessibility areas had lower odds of having BCS, while  the odds for women from middle (OR 0.81 (0.69, 0.94)) and most disadvantaged (OR 0.87 (0.71, 0.98)) areas was significantly lower than women living in affluent areas. The association between accessibility and the type of surgery reduced over time (interaction *p* = 0.028) but not for area disadvantage (interaction *p* = 0.209). In making informed decisions about surgical treatment, it is crucial that any geographical-related barriers to implementing their preferred treatment are minimised.

## 1. Introduction

Breast cancer is the most frequently diagnosed cancer and the leading cause of cancer mortality among females worldwide, accounting for 25% of total cancer cases and 15% of all cancer deaths in 2012 [1]. Current evidence-based recommendations for the management of early breast cancer state that when breast conserving surgery (BCS) is not clinically contraindicated, all women should be given an informed choice between BCS and mastectomy [2,3,4]. Although both procedures are considered clinically equivalent in terms of survival [5], BCS is less invasive and is associated with improved quality of life outcomes pertaining to body image, sexuality, self-esteem, and social adjustment [6,7,8,9,10,11]. Moreover, women receiving BCS were shown to have fewer post-operative complications than mastectomy [12,13]. Given ongoing improvements in survival, cosmetic and psychosocial outcomes following breast cancer treatment, may become increasingly important as a decisive factor during treatment decision-making.

Despite these guidelines, widespread geographical and socioeconomic variations in the clinical management and patterns of care for early stage breast cancer persist, in particular, rural and more disadvantaged women are consistently less likely to receive BCS [14,15,16,17,18,19,20,21,22,23,24] than their urban or less disadvantaged counterparts. These disparities are widely attributed to a combination of clinical, demographic, environmental, and healthcare system factors including geographical access to and availability of specialised care, comorbidities, and patient preferences [14,15,16,18,19,21,22,23,25,26,27,28,29,30].

Among Australian women, geographical variations in breast cancer patterns of care are well documented with studies based on the Breast Cancer Audit which includes about 60% of all early breast cancer cases diagnosed in Australia reporting that rural [16,22,23] and disadvantaged women [15] were more likely to undergo a mastectomy independent of the tumour size. Similar patterns were also found by several cancer-registry linkage studies [20,21,24].

Previously, we reported on the patterns of surgical treatment for women diagnosed with early breast cancer during 2004 in Queensland, Australia [24]. Here we update and extend that work to explore whether there is any evidence of changes in the likelihood of women being treated with BCS rather than mastectomy over the fifteen-year period between 1997 and 2011.

Various initiatives have been developed over the last decade to improve access to quality cancer care for patients in rural, remote and other disadvantaged settings in Australia such as establishment of regional cancer care centres [31,32] and innovative models of care [33,34]. However, to the best of our knowledge, the impact of such initiatives on geographical patterns of care for breast cancer, have not been previously explored at a population level.

## 2. Materials and Methods

Ethical approval to conduct this study was obtained from the Griffith University Human Research Ethics Committee (GU Ref No: PBH/34/13/HREC). Queensland Health gave legislative consent to access the routinely collected population-based dataset of incident breast-cancer cases, and to link it with the Queensland Hospital Admitted Patient Data Collection (QHAPDC) [35].

Data was obtained from the Queensland Cancer Registry (QCR) for all women aged at least 20 years diagnosed with a first and only primary histologically verified invasive breast cancer (ICD-O3 C50) between 1997 and 2011 [36] with known geographical information, tumour size, and lymph node involvement. By definition our exclusion criteria excluded any women with synchronous bilateral breast cancer at the time of initial presentation. Likewise, metachronous and recurrent breast cancers were also excluded. Only women with a first primary invasive breast cancer were considered as a prior cancer diagnosis may have impacted breast cancer treatment. Women with unknown tumour size or nodal information (2941, 8%) were also excluded. Information on hormone receptor status is not collected by the QCR. Variables extracted included age, year, partner status, Indigenous status (self-identified), breast cancer morphology, histology, tumour size, lymph node status, as well as information regarding any subsequent invasive primary (non-breast) cancers diagnosed according to the international rules for coding multiple primaries [37]. Women with no additional invasive (non-breast) cancers following their initial breast cancer were classified as “solitary” breast cancer cases.

In the absence of stage information, data collected since 1997 on tumour size and lymph node status [36] enabled a proxy measure of breast cancer stage at diagnosis to be calculated [38]. This study was restricted to women diagnosed with localised disease which was defined as tumours with a diameter of 20 mm or less with no evident nodal spread or metastases.

### 2.1. Treatment and Comorbidities

A deterministic link based on hospital and admitted patient number between the QCR and the QHDAPC database was used to obtain information on all breast-cancer related surgical procedures and comorbidities [24,39]. All women in the cohort were followed for up to two years after their breast cancer diagnosis consistent with previous studies [24,40], with the latest follow-up being 31 December 2013. Multiple records were possible for the same woman. Our comorbidity information was limited to those recorded in hospital charts during an inpatient episode of care within the two-year period following the breast cancer.

#### 2.1.1. Surgical Procedures

The cohort was restricted to women who had either a BCS or mastectomy during the study period. A woman was assumed to have undergone a specific procedure if it was identified on at least one linked hospital record. Prior to 1 July 1998 all surgical procedures were coded using ICD-9-CM codes; but from that date forward they were coded in ICD-10-AM [41,42]. Surgical procedures were categorised as BCS (ICD-9-CM code 85.20–85.23, ICD-10-AM block codes 1744–1745) or mastectomy (ICD-9-CM code 85.41–85.48, ICD-10-AM block codes 1747–1749) (see Table S1).

#### 2.1.2. Hospital Type

Each woman was allocated a hospital volume, based on the annual breast-cancer related surgical caseload of the hospital where they underwent surgery and also categorised as either public or private [35].

#### 2.1.3. Comorbidities

Hospital comorbidities are conditions that either co-existed with or arose during at least one breast-cancer related inpatient stay and which influenced a patient’s clinical management. Data on all comorbidities included in the Charlson Comorbidity Index were obtained based on diagnostic codes (see Table S1) [42]. The Charlson Comorbidity Index (CCI) score [42,43] was then collapsed to none (CCI = 0), low (CCI = 1), or moderate/severe (CCI ≥ 2). We excluded cancers from this list as we were unable to ascertain whether the recorded cancer was independent of the primary breast cancer diagnosis.

### 2.2. Geographical

Each woman was assigned a Statistical Local Area (SLA) according to the Australian Standard Geographical Classification [44] based on the geocoded coordinates of her residence at diagnosis. SLAs are administrative units that cover the whole of Queensland with no gaps or overlaps that are deemed to be socio-economically relevant to their residents and relatively homogeneous in terms of the population covered [38,44]. In 2011, the median population among 478 SLAs in Queensland was 6390 (range 2: 83,600). Women were then categorized into four accessibility groups (see Table 1) based on the road travel time from their residential SLA to the closest radiation facility [45]. Radiation facilities in Queensland are typically affiliated to major cancer care centres, hence these distances serve as a proxy measure of access to optimum oncological services and so better reflect access from the perspective of specialist cancer treatment facilities than more generic area-based classifications such as the Australian Accessibility/Remoteness Index [46].

Area-level socioeconomic status was measured according to the SLA-based Index of Relative Socioeconomic Advantage and Disadvantage (IRSAD) which was developed by the Australian Bureau of Statistics and ranks SLAs in Australia according to their relative socio-economic advantage and disadvantage [47]. The IRSAD summarises information about the socio-economic status of people and households within an SLA, comprising both relative advantage and disadvantage measures.

Women who were not matched to any hospital treatment records were excluded to avoid potential misclassification as we could not be certain if they had undergone treatment interstate, had no treatment, or if the missing information was due to errors in the data linkage processes [24,39]. Women with missing geographical information (less than 0.05% of cohort) were also excluded.

### 2.3. Analyses

Bivariate associations were explored using chi-squared tests. Multivariate logistic regression models were then used to identify key predictors of BCS as opposed to mastectomy, including checking that model assumptions such as lack of collinearity were met with appropriate diagnostic tests in Stata (StataCorp, College Station, TX, USA). No evidence for collinearity between variables was found when assessing the variance inflation factor. A stepwise model building process was used, in which all variables (Table 1) were initially entered into the model before sequentially removing those deemed non-significant (*p* > 0.20) to obtain the provisional reduced final model. Each of the dropped variables was then tested individually to determine whether their inclusion improved the model fit using the likelihood ratio test. Goodness of fit was also assessed graphically based on model residuals.

We examined whether the patterns for BCS by accessibility or residential disadvantage varied over time by separately including interaction terms between these explanatory variables and the year of diagnosis in the corresponding main effect model. Interaction terms were considered statistically significant if *p* ≤ 0. 05 (Wald χ^2^ test) for the overall effect.

Initial tests of bivariate associations (not shown) highlighted that the individual effects of the ordinal variables of age and year of diagnosis on the probability of having BCS were not linear. We explored various transformations, including restricted cubic splines. For age, we found there was little difference in model fit between categorizing age (categories in Table 1) and the cubic splines, so for ease of presentation age was included as a categorical variable. For year of diagnosis, the cubic splines provided the best fit with two degrees of freedom for the knots and have been used in the adjusted models.

All statistical analyses were performed with Stata/SE version 14 (StataCorp, College Station, TX, USA). Parameter estimates from main-effect models are presented as adjusted odds ratios (OR) with their 95% confidence intervals (CI). Since the parameter estimates for cubic splines for year of diagnosis have no interpretation in themselves, we calculated the predicted odds ratios, and their 95% CI, using the predicted probabilities from the model [48]. Likelihood ratio tests were used to assess the contribution of each variable to model fit, with statistical significance taken to be *p* ≤ 0.05.

The average adjusted predicted probabilities of having BCS for a specified combination of covariates were estimated from the fully adjusted interaction models and expressed as a percentage.

Sensitivity analyses using varying cut-points ranging from 50 to 200 cases per year to define high caseload hospitals and comparison of observed and predicted probabilities of undergoing BCS were used to guide our choice of most appropriate way of modelling this variable, with the final cut-points being 0–74, 74–99 and ≥100 cases per year.

Additional sensitivity analyses assessing the impact of unknown stage on observed associations were carried out by repeating the analyses assuming that all cases with unknown stage were localised or by randomly assigning them equally to localised and advanced categories.

## 3. Results

### 3.1. Cohort Characteristics

Of the 35,693 cases of invasive female breast cancer in Queensland between 1997 and 2011, 16,445 (46%) women who were diagnosed with localized disease aged 20 years and over and had known geographical information, initially comprised the study cohort. The exclusion of cases without a matching hospital record (*n* = 1399, 4%), a prior invasive cancer diagnosis (*n* = 918, 3%), who had both mastectomy and BCS (*n* = 1318, 4%) or a linked axillary staging procedure (*n* = 212, <1%) and those with multiple breast cancers (*n* = 967, 3%) gave a final cohort of 11,631 women. Of these, 3% lived in areas categorized as having very low accessibility and 12% in areas classified as being most disadvantaged (Table 1). The median age at breast cancer diagnosis was 59 years (interquartile range: 51–68 years).

Over half (56%) of the women had surgery at a private hospital, and 54% at a hospital with an annual caseload of at least 100 (high volume). However, these proportions varied by residential location, with women living in high and very highly accessibility areas or from more advantaged areas being significantly more likely (*p* < 0.001) to attend a high volume or private hospital.

### 3.2. Associations with Likelihood of Having BCS

Overall, 9223 women (79%) in the cohort underwent BCS and 2403 (21%) a mastectomy during the two-year of follow-up since diagnosis (Table 1). Over the study period (1997–2011), there was a statistically significant increase in the percentage of women undergoing BCS (*p* < 0.001) from around 70% in 1997 to 81% in 2000, after which it became relatively stable for the remainder of the study period (fluctuating between 78% and 82%).

All of the covariates considered in this study (Table 1) had a significant (log rank test: *p* < 0.001) bivariate association with BCS. After adjustment in the multivariable model, for some variables the strength of the association reduced, however all were significant at the 5% level.

After adjustment for socio-demographic and clinical factors, women who lived in high (OR 0.58 (95% CI 0.49, 0.69)), low (OR 0.47 (0.41, 0.54)) and very low (OR 0.44 (0.34, 0.56)) accessibility areas had significantly lower odds of having BCS than their counterparts from very high accessibility areas. Women from middle (OR 0.81 (0.69, 0.94)) or most disadvantaged (OR 0.87 (0.71, 0.98)) areas also had lower odds of BCS than residents of least disadvantaged areas. Other independent predictors of lower uptake of BCS included higher grade, comorbidities or a subsequent diagnosis of another primary invasive (non-breast) cancer. By contrast the odds of women having BCS at a high caseload (≥100 versus <75, OR 1.58 (95% CI 1.41, 1.78)) or a private (versus public, OR 1.48 (1.33–1.64)) hospital were significantly higher (Table 1).

### 3.3. Interaction Models

**Figure 1:**
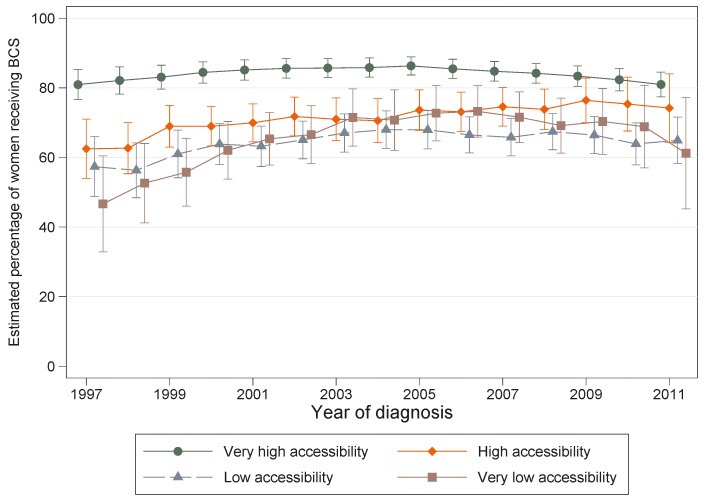
Estimated number of women with localised breast cancer out of 100 (percentage) undergoing breast conserving surgery (BCS) by accessibility and year of diagnosis, Queensland 1997–2011.

The interaction between the effects of accessibility and year of diagnosis on the likelihood of having BCS was statistically significant (*p* = 0.028). Although the estimated percentage of women from very high accessibility areas having BCS was consistently higher than the corresponding percentages for high, low and very low accessibility areas for each year of diagnosis, the magnitude of these differences narrowed over time (Figure 1). For example, these percentages increased slightly over time from 47% (1997) to 61% (2011) for women living in very low accessibility areas whereas they remained around 81% for women from very high accessibility areas. The non-overlapping confidence intervals for the very low or low accessibility areas and those of the very high accessibility areas highlighted that the significant geographical difference in the likelihood of BCS remained over the whole study period.

**Figure 2:**
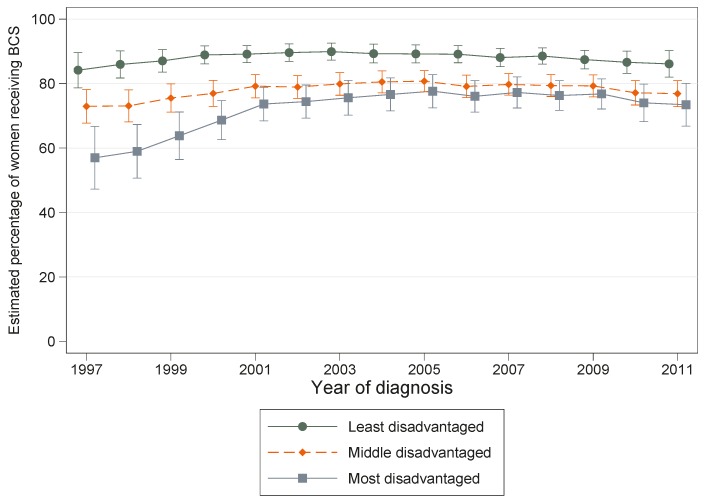
Estimated number of women with localised breast cancer out of 100 (percentage) undergoing BCS by residential disadvantage and year of diagnosis, Queensland 1997–2011.

While there was a suggestion of converging patterns in the percentages of women receiving BCS by area disadvantage during the late 1990s (Figure 2), these curves had plateaued since 2000, meaning that overall there was no statistical evidence that the association between area disadvantage and likelihood of BCS (interaction *p* = 0.209) had changed over time.

## 4. Discussion

This study was designed to examine temporal changes in geographical patterns of BCS by residential location as measured by accessibility and area-disadvantage. Among women diagnosed with localised breast cancer in Queensland, Australia, we found that the likelihood of receiving BCS as opposed to mastectomy varied by geographical location. In particular, after adjusting for other variables, women living in low and very low accessibility areas were significantly less likely to undergo BCS than those living in very high accessibility areas. Women from disadvantaged areas were also less likely to undergo BCS than their counterparts living in more affluent areas. Our results were consistent with other Australian studies that reported lower uptake of BCS among women living in more remote or disadvantaged areas [15,16,20,21,22,23,24].

However, to the best of our knowledge ours is the first study to investigate whether these geographical patterns have changed over time. Given ongoing improvement in the provision of cancer care in regional Australia over the past decade, this type of study can serve to examine the potential impact of the addition of these services on patterns of cancer care and may provide a benchmark for future studies in this area. While we found statistical evidence of a change in these patterns for women diagnosed over a period of 15 years, in practice the changes were slight and in the most recent year of diagnosis women from areas of lower accessibility or more disadvantage were still less likely to undergo BCS than those living in highest accessibility or least disadvantaged areas. It remains to be seen whether they are suggestive of a definite change in geographical inequalities.

Australian clinical practice guidelines for early breast cancer management stipulate that where clinically appropriate, women should be offered the choice of either BCS with adjuvant radiotherapy, or mastectomy [4], as curative surgical treatment, as both are considered clinically equivalent in terms of survival [5]. However, women should also be informed that body image is better preserved with BCS during the treatment decision-making process [4]. Given the well-documented inequitable cancer care across population-subgroups by geographical location in Australia [15,16,22,23,24], our evidence for a reduction in geographical disparities in patterns of surgical treatment (after adjustment) is supportive of some reduction in the impact of distance-related geographical barriers to accessing cancer care. Importantly, since distance is not only a barrier to access in itself but contributes to disproportionate financial costs [49], and psychosocial outcomes [50] of breast cancer patients needing to travel for treatment, this evidence is encouraging.

In most cases, adjuvant radiotherapy is recommended with BCS to reduce the risk of long-term recurrence for women diagnosed with breast cancer [3,4]. Thus our finding that women who lived in less accessible areas were less likely to have BCS is not surprising, and is consistent with other studies showing that women living in rural areas or further away from radiotherapy facilities are less likely to undergo BCS and adjuvant radiotherapy following BCS across Australia [15,21,22,51] and the United States [18,19,26,52]. The following scenarios would indicate an inequity in access to care; first, when women from areas of less accessibility were not offered the opportunity to make an informed decision regarding BCS versus mastectomy where it was clinically appropriate, or second, when women had a mastectomy solely due to difficulties in accessing radiotherapy services even if they preferred BCS. A recent Australian study highlighted how access to treatment impacts on treatment choice, with a greater proportion of women diagnosed with breast cancer having BCS following the opening of a local publicly funded radiotherapy centre in their local area [53]. While there has been an expansion of radiotherapy infrastructure in regional centres since 2011 [32], the last year of our study cohort, our categories of accessibility have taken that into account, so this expansion cannot explain the reducing impact of distance on the choice of surgical treatment. Previously, it was suggested that a surgeon’s preference had been a factor in this disparity [54,55], however the current study shows that most patients were treated in high volume centres, making this less likely. Specialisation, as measured by annual breast cancer surgical caseload can influence surgical treatment with lower-caseload surgeons being more likely to prefer a mastectomy [54,55,56]. Treatment at high volume hospitals with site-specific multidisciplinary teams and specialised surgeons with expertise in performing BCS is however an independent predictor of BCS with adjuvant radiotherapy [23,24,55,56,57]. Other confounding influences on BCS rates such as the rise of “oncoplastic surgery” [58], and the acceptance of narrower margins of excision in attempting BCS [59] are more relevant to the time period immediately after the 2011 cohort.

Other contributing factors to the narrowing geographical disparities over time may include improved access to specialist services and breast cancer surgeons through establishment of regional cancer centres [31,32], the adoption of novel approaches to improve both the efficiency and quality of cancer services for people in less accessible settings (such as telehealth and integrated service networks) [33,34] as well as changes in perceptions of BCS versus mastectomy. We lack the information in our study cohort to explore these issues further. Studies from Australia [15,16,20,21,24,25], Europe [14,27], Canada [17], and the United States [18,19,26] have however consistently reported associations between sociodemographic, clinical, geographical and health-care factors and patterns of surgical care for early breast cancer suggesting that there are other factors, apart from accessibility and area disadvantage, that drive the choice of BCS versus mastectomy.

We found no evidence for a significant reduction in the disparities of BCS by residential disadvantage over time. Our measure of residential disadvantage includes both relative advantage and disadvantage measures such as low and high educational attainment, unemployment, occupation type, as well as low and high income [47] and is based on SLAs that are deemed relatively homogenous in terms of the covered populations [44]. The underlying reasons for the persistent variations in surgical patterns by residential disadvantage are unclear. The QCR [36] does not collect information on potential confounders such as measures of individual socioeconomic status including income, education, and health insurance status [15,19,52,60,61]. Unmeasured variations in general health status at time of breast cancer diagnosis is another possibility as more disadvantaged women are known to have poorer health and a higher prevalence of comorbidities [62] which may influence treatment decisions [30]. More disadvantaged women may also experience greater financial and structural barriers to accessing specialised care and/or high-volume hospitals [63,64,65] both of which are independent predictors of care in accordance to guidelines (and higher receipt of BCS) [23,24,55,56,57].

Treatment-decision making is a complex procedure which is impacted by choices offered to a patient by their clinician and patient preferences. Clinicians typically consider factors such as comorbidities, age, clinical characteristics, and ability to tolerate adjuvant therapy when recommending treatment [2]. Two recent reviews [28,66] suggested that a mix of issues, including those related to body image, fertility, concerns about survival or recurrence, quality of life, and clinician preferences were key drivers for a woman’s preference of surgical treatment and adjuvant therapies for early breast cancer. In one review, Hamelinck and colleagues [28] found that, for some women, survival was a decisive factor in their preference for mastectomy over BCS, which is of concern given that both approaches have clinically equivalent survival [5], suggesting existing limitations in the effective communication of medical information that adequately explains the risks and benefits of both approaches.

To the best of our knowledge, no Australian study has explored how the provision of medical information by clinicians to women diagnosed with breast cancer varied by geographical location, and whether there is any differential impact of this information on patient choice. It remains a priority to ascertain how the provision of information to patients impacts on the decision making process, and how this can be improved by health care providers and other support personnel. However, it is one thing to ensure that all women are provided information about the possible surgical options following a diagnosis of breast cancer, it is another thing to ensure that all the options discussed are available to women regardless of where they live. With the decentralised population in Queensland, and large geographical distances, efforts to ensure equitable access to services in all areas will continue to be an issue.

### Strengths and Limitations

The strengths of this study include the use of 15 years of diagnostic data from a robust state-wide population-based cancer registry to which notification of all invasive breast cancers diagnosed in Queensland is a statutory requirement [36]. Our population-based data linkage approach, using a deterministic linkage method, also allowed us to identify breast-cancer related surgical procedures within two years of diagnosis at all public and private hospitals within Queensland and selected hospital non-cancer comorbidities.

However, important limitations remain including our inability to adjust for more detailed patient socio-demographics, lifestyle and psychosocial variables, and important clinical factors such as hormone receptor status [22,23,25], family history [51], and adjuvant therapies [21,22,25] that may have influenced treatment decisions but are not collected by the accessed administrative databases. Information on surgical status and selected comorbidities were obtained through a deterministic linkage between the QCR and QHAPDC. As such, this data would be missing for women who received subsequent treatment interstate or overseas [39]. We acknowledge that some instances of surgery may also have been missed due to errors in the notification or data linkage process [24,40].

Our comorbidity information was limited to those within two years of diagnosis that were considered by hospital staff to have directly impacted on treatment, and so may have been underestimated. We were unable to assess the influence of pre-existing comorbidities on breast-cancer related treatment decisions, however international studies report that pre-existing conditions have tended to be associated with an increased likelihood of receiving mastectomy [30].

Around 8% of cases could not be staged and were excluded from the analyses. However, sensitivity analyses showed that the association between decreasing accessibility, increasing disadvantage, and lower rates of BCS remained, regardless of the assumptions made about the true distribution of unknown stage cancers. Given their very small numbers, the exclusion of women lacking geographical information is unlikely to have any impact on results.

## 5. Conclusions

Our results suggest that distance as a barrier to accessing treatment may have reduced over time. Since we are limited in our ability to examine in depth the underlying reasons for these changes, both the drivers of the geographical variations in surgical patterns by area disadvantage and the temporal trends in these patterns remain unclear, although environmental, structural, social, and economic factors remain plausible explanations. As such although the temporal reduction in the geographical variation in the type of surgical care for women diagnosed with localised breast cancer is encouraging, further research to identify the drivers of existing disparities, especially those related to health care system, area disadvantage, provision of adequate information and decision making factors remains a priority. It is crucial that all women diagnosed with breast cancer not only have the opportunity to make informed decisions about their care and treatment in partnership with clinicians, but that any geographical-related barriers to implementing their preferred treatment are minimised.

## Figures and Tables

**Table 1:** Cohort of women with a first primary localised breast cancer, Queensland 1997–2011 having breast conserving surgery.

Variable	Numbers	Breast Conserving Surgery (BCS)	
	N (col %)	N (%) Having BCS	Adjusted Odds Ratio (BCS: Mastectomy with 95% Confidence Interval in Brackets) ^2^
Number of women in cohort	11,631 (100.0)	9223 (79.3)	
*Residential disadvantage*		*p* < 0.001 ^1^	*p* = 0.013 ^3^
Least disadvantaged	2044 (17.6)	1797 (87.9)	1.00
Middle socioeconomic status	8237 (70.8)	6440 (78.2)	0.81 (0.69, 0.94)
Most disadvantaged	1350 (11.6)	986 (73.0)	0.87 (0.71, 0.98)
*Accessibility*		*p* < 0.001	*p* < 0.001
Very high (<1 h)	8523 (73.3)	7157 (84.0)	1.00
High (1–2 h)	932 (8.0)	653 (70.1)	0.58 (0.49, 0.69)
Low (2–6 h)	1817 (15.6)	1179 (64.9)	0.47 (0.41, 0.54)
Very low (≥6 h)	359 (3.1)	234 (65.2)	0.44 (0.34, 0.56)
*Age at diagnosis (years)*		*p* < 0.001	*p* < 0.001
<40	586 (5.0)	466 (79.5)	0.80 (0.63, 1.01)
40–49	1969 (16.9)	1613 (81.9)	0.86 (0.74, 1.01)
50–59	3277 (28.2)	2755 (84.1)	1.00
60–69	3336 (28.7)	2656 (79.6)	0.77 (0.68, 0.88)
70–79	1871 (16.1)	1349 (72.1)	0.53 (0.45, 0.61)
≥80	592 (5.1)	384 (64.9)	0.40 (0.32, 0.49)
*Year of diagnosis ^4,5^*		*p* < 0.001	*p* = 0.002
1997	507 (4.4)	353 (69.6)	1.00
1998	594 (5.1)	443 (74.6)	1.03 (0.99, 1.07)
1999	632 (5.4)	486 (76.9)	1.17 (1.13, 1.21)
2000	678 (5.8)	549 (81.0)	1.27 (1.22, 1.32)
2001	668 (5.7)	540 (80.8)	1.48 (1.43, 1.54)
2002	705 (6.1)	576 (81.7)	1.49 (1.43, 1.54)
2003	704 (6.1)	565 (80.3)	1.55 (1.49, 1.61)
2004	744 (6.4)	594 (79.8)	1.57 (1.51, 1.62)
2005	788 (6.8)	646 (82.0)	1.63 (1.57, 1.69)
2006	838 (7.2)	687 (82.0)	1.51 (1.46, 1.56)
2007	792 (6.8)	634 (80.1)	1.53 (1.48, 1.59)
2008	927 (8.0)	736 (79.4)	1.49 (1.44, 1.54)
2009	1009 (8.7)	789 (78.2)	1.48 (1.43, 1.52)
2010	1040 (8.9)	825 (79.3)	1.32 (1.28, 1.36)
2011	1005 (8.6)	800 (79.6)	1.30 (1.26, 1.34)
*Partner ^6^*		*p* < 0.001	*p* = 0.036
Partnered	7427 (63.9)	6003 (80.8)	1.00
No partner	3937 (33.8)	2990 (75.9)	0.94 (0.84, 0.96)
Unknown	267 (2.3)	230 (86.1)	1.40 (0.95, 2.06)
*Indigenous*		*p* < 0.001	*p* = 0.045
Non-Indigenous	10,177 (87.5)	7993 (78.5)	1.00
Indigenous	87 (0.7)	62 (71.3)	0.96 (0.71, 1.78)
Unknown	1367 (11.8)	1168 (85.4)	1.23 (1.02, 1.47)
*Histology*		*p* < 0.001	*p* < 0.001
Ductal (8500-3)	8994 (77.3)	7168 (79.7)	1.00
Lobular (8520-3)	1094 (9.4)	808 (73.9)	0.73 (0.62, 0.85)
Other	1543 (13.3)	1247 (80.8)	1.11 (0.96, 1.28)
*Size (mm)*		*p* < 0.001	*p* < 0.001
<10 mm	3905 (33.6)	3260 (83.5)	1.00
10–20 mm	7726 (66.4)	5963 (77.2)	0.75 (0.67, 0.83)
*Grade*		*p* < 0.001	*p* <0.001
Low	3391 (29.2)	2829 (83.4)	1.00
Intermediate	4754 (40.9)	3727 (78.4)	0.76 (0.67, 0.86)
High	2370 (20.4)	1839 (77.6)	0.67 (0.58, 0.77)
Unknown	1116 (9.5)	828 (74.2)	0.63 (0.52, 0.76)
*Multiple primary cancers ^7^*		*p* < 0.001	*p* <0.001
No	10,941 (94.1)	8760 (80.1)	1.00
Yes	690 (5.9)	463 (67.1)	0.53 (0.44, 0.64)
*Comorbidity ^8^*		*p* < 0.001	*p* = 0.022
None	11,027 (94.8)	8807 (79.9)	1.00
Low	491 (4.2)	347 (70.7)	0.76 (0.61, 0.95)
Moderate/severe	113 (1.0)	69 (61.1)	0.62 (0.41, 0.94)
*Hospital type*		*p* < 0.001	*p* <0.001
Public	5106 (43.9)	3834 (75.1)	1.00
Private	6525 (56.1)	5389 (82.6)	1.48 (1.33, 1.64)
*Hospital volume ^9^*		*p* < 0.001	*p* <0.001
0–74	3761 (32.3)	2763 (73.5)	1.00
75–99	1636 (14.1)	1121 (68.5)	0.78 (0.68, 1.02)
≥100	6234 (53.6)	5339 (85.6)	1.58 (1.41, 1.78)

^1^ *p* values from Pearson’s chi square test; ^2^ Models adjusted for all covariates; ^3^ *p*-values from Wald’s joint test of coefficients for multivariate logistic regression; ^4^ Year was modelled as a continuous variable with restricted cubic splines. ^5^ The parameter estimates for cubic splines for year of diagnosis have no interpretation in themselves, hence predicted odds ratios and their 95% CI were calculated using the predicted probabilities from the model ^6^ Partner includes married and partnered; no partner includes never married/single, widowed, separated and divorced; ^7^ Other invasive cancers following breast cancer; ^8^ Based on Charlson Comorbidity Index (CCI) score: none (CCI = 0), low (CCI = 1) and moderate/severe (CCI ≥ 2); ^9^ Annual breast cancer surgical caseload.

## Supplementary Materials

The following are available online at www.mdpi.com/1660-4601/13/7/729/s1, Table S1: ICD-9-CM and ICD-10-AM procedure and disease codes.

## Abbreviations

The following abbreviations are used in this manuscript:

BCS	Breast conserving surgery
QHAPDC	Queensland Hospital Admitted Patient Data Collection
QCR	Queensland Cancer Registry
CCI	Charlson Comorbidity Index
SES	Socioeconomic status
SLA	Statistical Local Area
OR	Odds Ratios
CI	Confidence Intervals
ICD-9-CM	International Classification of Diseases, 9th Revision, Clinical Modification
ICD-10-AM	International Classification of Diseases, 10th Revision, Australian Modification
