# Supplementary material for: Geographical Inequalities in Surgical Treatment for Localized Female Breast Cancer, Queensland, Australia 1997–2011: Improvements over Time but Inequalities Remain
Source: Int J Environ Res Public Health. 2016 Jul 19;13(7):729. doi: 10.3390/ijerph13070729 (PMC4962270; doi:10.3390/ijerph13070729)
Supplement: Supplementary file 1 [file ijerph-13-00729-s001.pdf]

Supplementary Materials: Geographical Inequalities in Surgical Treatment for Localized Female Breast Cancer, Queensland, Australia 1997–2011-Improvements over Time but Inequalities Remain

Peter D. Baade, Paramita Dasgupta, Philippa H. Youl, Christopher Pyke and Joanne F. Aitken

Table S1. ICD-9-CM and ICD-10-AM procedure and disease codes.

Type of Surgery/Disease	ICD-9-CM Codes	ICD-10-AM Codes
Breast cancer related procedures		
Breast conserving surgery	85.20–85.23	**Blocks (1744–1745)**<br>30342-00, 30342-01, 30346-00, 30346-01, 30347-00, 30348-00, 30350-00, 30350-01, 31500-00, 31515-00.
Mastectomy	85.41–85.48	**Blocks (1747–1749)**<br>30338-00, 30338-01, 30338-02, 30351-00, 30351-01, 30353-00, 30353-01, 30353-02, 30354-00, 30354-01, 30356-00, 30356-01, 31518-00, 31518-01, 31524-00, 31524-01, 30359-00, 30359-01, 30359-02, 30359-03, 30359-04, 30359-05, 30359-06, 30359-07
Comorbidities		
Myocardial infarction	410, 412	I21–I22, I25.2
Congestive heart failure	428	I50
Peripheral vascular disease	441; 442–443	I71; I73; I79.0
Cerebrovascular disease	430–432	I60–I64
Dementia	290	F00–F01
Chronic pulmonary disease	490–496; 500–505	J40–J47; J60–J67
Rheumatologic disease	710.0–710.1; 710.4; 714.0–714.2; 725	M32; M33.2; M34; M35.3; M05.0–M05.3; M05.8–M06.0; M06.3; M06.9
Peptic ulcers	531–534	K25–K28
Liver disease (mild)	571.2; 571.4–571.6	K70.2–K70.3; K71.7; K74.0; K74.2–K74.6
Diabetes (without complications)	250.1–250.3; 250.7	E10.1; E10.5; E10.9; E11.1; E11.5; E11.9; E13.1; E13.5; E13.9; E14.5; E14.9
Diabetes (with complications)	250.4–250.6	E10.2–E10.4; E11.2–E11.4; E13.2–E13.4; E14.2–E14.4
Hemiplegia/Paraplegia	342; 344.1	G04.1; G81; G82.0–G82.2
Renal disease	580–588	N00; N03–N05; N07.2–N07.4; N17–N19
Liver disease (moderate/severe)	572.2–572.4; 572.8	K72.1; K72.9; K76.6–K76.7
AIDS/HIV [1]	042–044	B20–B24

[1] AIDS: Acquired immunodeficiency syndrome; HIV: human immunodeficiency virus.

© 2016 by the authors; licensee MDPI, Basel, Switzerland. This article is an open access article distributed under the terms and conditions of the Creative Commons by Attribution (CC-BY) license (http://creativecommons.org/licenses/by/4.0/).
